# Effect of Depositional Environment on the Occurrence of 17α(H)-Diahopanes in Triassic Lacustrine Sediments from the Ordos Basin, NW China

**DOI:** 10.1021/acsomega.4c06057

**Published:** 2024-11-05

**Authors:** Baohong Shi, Quansheng Liang, Erhu Liu, Xinyu Ai, Rong Wang

**Affiliations:** † School of Earth Sciences and Engineering, Xi’an Shiyou University, Xi’an, Shaanxi 710065, P. R. China; ‡ Shanxi Key Laboratory of Petroleum Accumulation Geology, Xi’an Shiyou University, Xi’an, Shaanxi 710065, China; § Research Institute of Yanchang Petroleum (Group) Co. Ltd, Xi’an, Shaanxi 710075, China; ∥ Gas Field Company, Shaanxi Yanchang (Group) Co. Ltd, Yan’an, Shaanxi 716000, China; ⊥ Natural Gas Research Institute, Shaanxi Yanchang Petroleum (Group) Company Limited, Xi’an, Shaanxi 710075, China

## Abstract

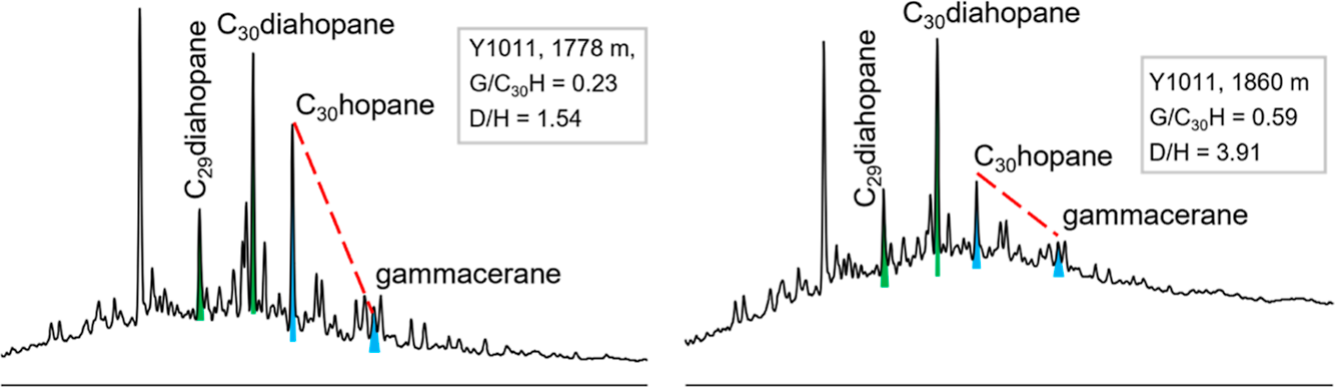

In this study, an abundant 17α(H)-diahopanes has been observed in the sediments from the seventh member of the Triassic Yanchang Formation in the central part of the Ordos Basin. The results show that the maturity has a minor contribution to the relative abundance of 17α(H)-diahopanes as the maturity of the samples are similar and fall into a limited range in the selected samples from the study area. The C_27_–C_29_ regular steranes distributions of the samples are also similar and shaped a “V” pattern, indicating a mixture of aquatic organisms and terrigenous organic matter, which may be not responsible for the different 17α(H)-diahopanes distributions. Mineral compositions indicate clay present in those samples with a distinctly enhanced abundance, which likely suggests that catalysis plays a critical role on the formation of arranged hopanes. Moreover, the relative contents of 17α(H)-diahopanes appear to be affected by the depositional environment due to a positive correlation with those of gammacerane, indicating that a more reducing water body is more helpful for the formation of 17α(H)-diahopanes, which is different from the conclusions in the previous studies. Therefore, the biomarker 17α(H)-diahopane may be an effective indicator for oil–oil/source correlation in this study area.

## 1 Introduction

The biomarker hopane series widely occurred in sediments and petroleum^1^ and have been employed for organic matter source, maturity, and depositional environment definition,^2,3^ as well as oil–oil/source correlation.^4,5^ The 17α(H)-diahopane, a series of rearranged hopanes, was first detected in petroleum by Moldowan et al. (1991)^6^ using X-ray crystallography and gas chromatography mass spectrometry/mass spectrometry (GC–MS/MS), and was suggested to be formed resulting from the catalytic rearrangement of hopenes at the stage of early diagenesis. The origin of 17α(H)-diahopanes is debated. These compounds, in earlier findings, have been considered to be terrestrial indicators as they are present in terrigenous petroleum and coals.^7−11^ However, the biological precursors of 17α(H)-diahopanes still remain in debate. The 17α(H)-diahopanes were first considered to be sourced from bacteria due to the similarity of stable carbon isotope composition with hopanes and their distribution as an extended pseudohomologous series.^6^ In addition, rhodophytes also can contribute to the occurrence of C_30_ diahopane.^12^ Algae may be also the origin of rearranged hopanes in lacustrine crude oils from Songliao Basin.^13^ Moreover, some bacterial communities living in a specific water body has been also proved to be the precursors of the rearranged hopanes.^14^ Numerous reports have suggested that an oxic and/or suboxic environment is beneficial to the formation of rearranged hopanes.^6,8,11,14−21^ However, a positive relationship between the relative abundances of 17α(H)-diahopane and gammacerane was observed in rocks and oils from Sichuan Basin, which indicates that a saline environment may promote the formation of this series of compounds.^22^ A similar relationship between the relative abundances of 17α(H)-diahopane and gammacerane was also observed in crude oils from Songliao Basin.^13^ Therefore, the effect of sedimentary environment on the distributions of 17-diahopanes is controversial.^23^ The mineral compositions of sediments in geological records have a significant influence on 17-diahopanes. The contents of clay minerals may likely to be the primarily controlling factor for the presence of rearranged hopanes.^24^ Maturity appears to be a significant factor controlling the relative abundance of 17α(H)-diahopane in the source rock and crude oils.^6,15,25,26^ The content of 17α(H)-diahopane in mudstones from lacustrine basin gradually increases with the increasing of buried depth^27^ and therefore is an effective indicator for maturity assessment for oil and source rock and petroleum system study. The ratios of 17α(H)-diahopane and regular hopane generally are applied for maturity assessment due to the relative thermostability to regular hopanes.

The 17α(H)-diahopanes in the sediments and oils from the Ordos Basin have been reported by numerous authors.^28−33^ Lithology appears to have a distinct effect on the 17-diahopanes distributions. The relative abundances of dishopane are various in the cores with different lithology,^30^ where dark mudstones formed under a suboxidizing water body are characterized by high content of rearranged hopanes while the carbonaceous mudstone, silty mudstone, and oil shale deposited on an anoxic environment contain little these compounds.^28,30^ However, depositional conditions and organic matter source control the occurrence of 17-diahopanes. Abundant 17α(H)-diahopane in the coal-bearing source rocks in the Ordos Basin likely is attributed to the input of terrigenous organic matter under a swamp environment.^29,32^ However, study on the main controlling factors for the formation of 17α(H)-diahopanes in different depositional areas are limited.

From the aforementioned related review, the influence of sedimentary conditions on the distributions of 17α(H)-diahopanes in organic matter remains debated. In this study, the sediments from the seventh member of the Triassic Yanchang Formation in the central area of paleolake of the Ordos Basin were systematically sampled from two wells and used to investigate the effect of the depositional environment on the distributions of 17α(H)-diahopane using GC–MS tool.

## 2 Geological Setting and Samples

**Figure 1:**
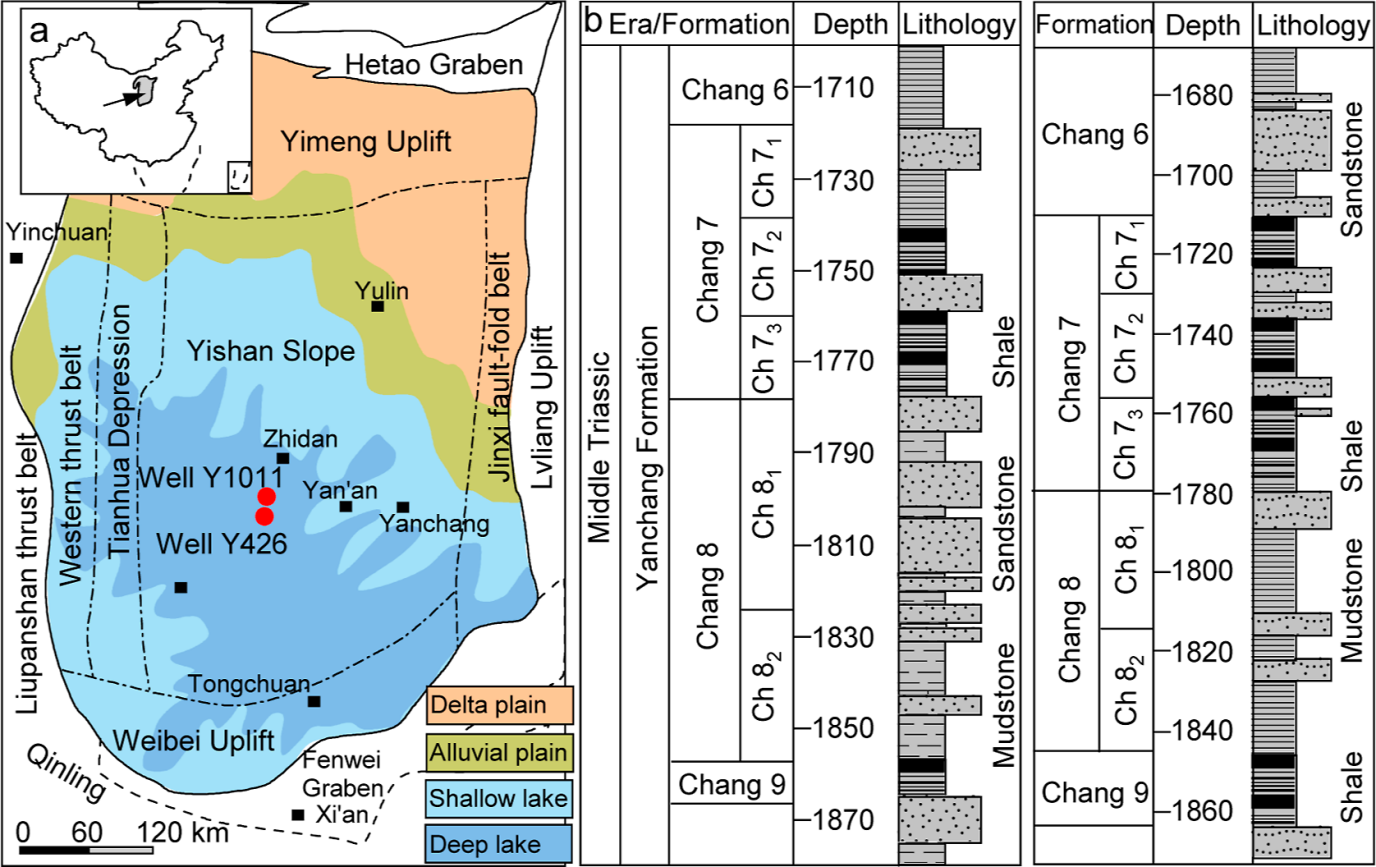
(a) Location and geotectonic divisions of the Ordos Basin showing the locations of sampled wells and the topography of the Ordos Basin showing the distributions of sedimentary environment and (b) lithologic profiles of Lower-Yanchang Formation (Chang 9–Chang 7).

As a stable tectonic unit in the North China Plate, the Ordos Basin developed above a metamorphic basement which had undergone a set of terrestrial lake filling during Late Triassic covering about 10 × 10^4^ km^2^.^34^ A total of six secondary structural units can be divided drawing upon the structural attributes of the Ordos Basin:^35−38^ Weibei uplift, Yimeng uplift, Yishan Slope, Jinxi fault and fold belt, Tianhuan depression, and western thrust belt (Figure 1).

The depositional environment evolved into lacustrine sedimentary as the response of the Indosinian Qinling tectonic activity in Late Triassic epoch which led to the accumulation of clastic depositional systems.^39^ The Yanchang Formation is recognized as the main oil-bearing strata within the Mesozoic of the Ordos Basin, which can be classified into 10 members from the bottom to top, respectively (Chang 10–Chang 1).^40^ As the most important source rocks, Chang 7 member deposited during the maximum flooding period in Late Triassic, which is characterized by the black extraordinarily high organic matter shale.^41^

In this study, a total of 32 core samples were collected from wells Y1011 and Y426 in the central paleolake of the Ordos Basin (Figure 1a). The samples from seventh member and ninth member of Triassic Yanchang Formation are black shale renown as “Zhangjiatan Shale” and “Lijiapan Shale” (Table S1, Figure 1b,c), which are important source rocks for the major discovered oils. Eight cores were from eighth member of Yanchang Foramtion characterized by dark gray mudstones (Table S1, Figure 1b,c), which is considered to be secondary source rocks for a few reservoirs trapped in the same beds.

## 3 Methods and Experiments

### 3.1 Total Organic Carbon Content Measurement

Rock samples studied were ground into less than 80 mesh in a crusher. Next, the powder sample was treated with diluted hydrochloric acid to remove the carbonates and then washed by deionized water for 48 h to remove remaining contaminants prior to analysis. The total organic carbon (TOC) content of the selected core samples was carried out on a LECO CS-230 Analyzer following the standard procedure.^38^

### 3.2 Rock-Eval Pyrolysis Analysis

About 100 mg of powdered samples was analyzed using an OGE-II equipment to obtain Rock-Eval pyrolysis related parameters, whose temperature programming involves that an initial temperature was set as 300 °C held for 3 min to get volatile hydrocarbon content and then heated to 600 °C with a rate of 25 °C/min to get remaining hydrocarbon content in the samples.

### 3.3 Gas Chromatography–Mass Chromatography (GC–MS)

Approximately 50 g of powder samples was extracted using a mixture of dichloromethane solvent to obtain the extractable organic matter (EOM). EOM was deasphaltened by about 50 mL of petroleum ether and then subdivided into three fractions, i.e., saturated hydrocarbon, aromatic hydrocarbon, and resin with 60 mL of petroleum ether, 40 mL of a mixture of dichloromethane and petroleum ether (2:1, v/v), and a mixture of dichloromethane and methanol (93:7, v/v), respectively, using column chromatography.

The GC–MS analysis of saturated hydrocarbons was performed in a TSQ 8000 Evo Triple Quadrupole GC–MS system equipped with an HP-5MS fused silica capillary column (60 m × 0.25 mm × 0.25 μm). The initial temperature of the GC system was 50 °C (held 1 min) and then programmed to 120 °C at a rate of 20 °C/min and next to 250 °C at a rate of 4 °C/min, finally ramped to 310 °C at a rate of 3 °C/min (held 30 min). The carrier gas was helium (purity >99.999%), and its rate was 1 mL/min. The injector temperature was 300 °C. The MS operating conditions were as follows: electron impact mode, 70 eV ionization energy, 100 μA filament current, 1200 V electron-multiplier voltage, and a scan range of 50–600 Da.

## 4 Results

### 4.1 Bulk Geochemical Characteristics

Table S1 lists the geochemical profiles of cores from wells Yong1011 and Yong426 to show the hydrocarbon generating potential. The TOC values range from 0.52% to 11.77% with an average of 4.17%. The *S*_1_ (volatile hydrocarbon content) values are within the range 0.29–8.87 mg/g (2.94 on average). The measured *S*_2_ (remaining hydrocarbon generative potential) range in the selected samples is from 0.21 to 32.58 mg/g with a mean of 11.71 mg/g. The *T*_max_ (temperature at maximum generation) values and vitrinite reflectance of the studied samples range in 440–459 °C and 0.79–0.91% Ro, respectively, indicating that all samples were at the oil-generated window.^42^ Therefore, these data show that most of the selected cores can be considered to be effective source rocks.^3,42,43^

### 4.2 Distributions of Diahopanes

**Figure 2:**
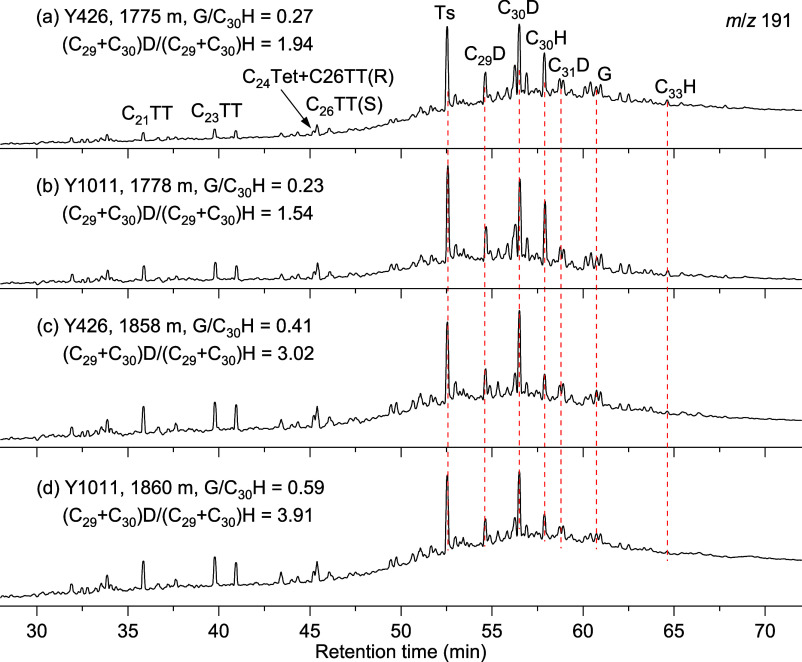
*m*/*z* 191 mass chromatograms showing the distributions of diahopanes in Chang 7 and Chang 9 formations. D: diahopane; H: hopane; and G: gammacerance.

An abundant 17α(H)-diahopane (Figure 2) has been observed in the rock extracts, where the member of C_30_ is dominant followed by the member of C_29_. The C_30_ 17α(H)-diahopanes have also been detected, but the compounds with higher carbon number are lower than the detection limits. The contents of 18a(H)-neohopanes (Ts) are distinctly low than those of 17α(H)-diahopane. The ratios of C_29_ + C_30_ 17α(H)-diahopane and C_29_ + C_30_ hopanes range from 0.10 to 4.58 (Table S1).

### 4.3 Distributions of *n*-Alkanes, Hopanes, and Steranes

The abundance of *n*-alkanes is much higher than that of isoprenoids, as shown in Figure S1. Relatively low values of isoprenoids over *n*-alkanes, i.e., pristane/*n*-C_17_ and phytane/*n*-C_18_ falling into 0.10–0.41 and 0.10–0.34, respectively (Table S1), were observed in the analyzed core samples. The Pr/Ph ratios range from 0.72 to 1.36, with a mean of 0.95 (Table S1).

The gammacerane indexes (Ga/C_30_H: gammacerane/C_30_hopane) of the sediments vary between 0.03 and 0.86, suggesting a fresh to brackish water body. The ratios of C_24_Tet/C_26_TT ranges from 0.22 to 0.41 (Table S1).

**Figure 3:**
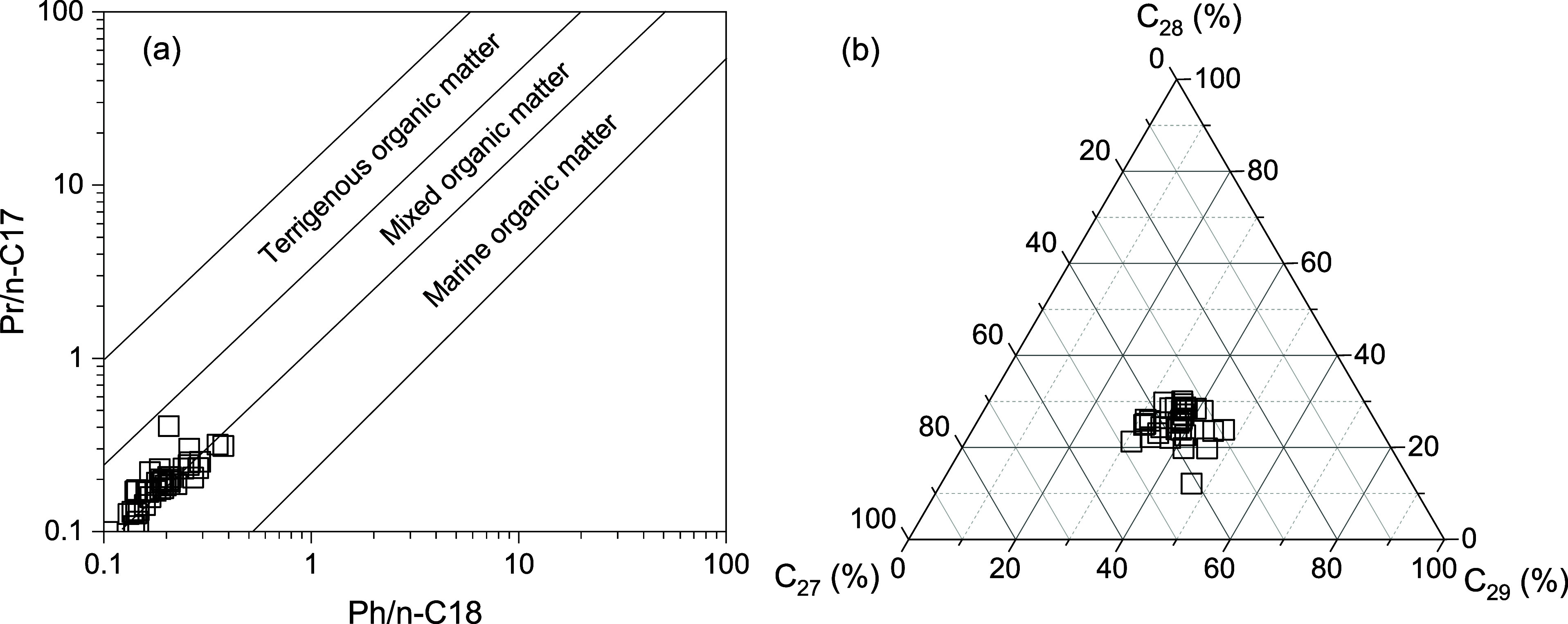
(a) Cross-plot of Ph/*n*-C_18_ and Pr/*n*-C_17_ and (b) ternary diagram showing the relative abundance of C_27_–C_28_–C_29_ regular steranes. Pr: pristane; Ph: phytane; C_27_(%): C_27_/(C_27_–C_29_) regular sterane; C_28_(%): C_28_/(C_27_–C_29_) regular sterane; and C_29_(%): C_29_/(C_27_–C_29_) regular sterane.

The C_27_–C_29_ regular sterane distributions of studied samples are similar and shaped a “V” pattern. The relative abundance of C_27_ and C_29_ regular steranes occur in a range of values from 29.24% to 47.76% and 31.00% to 47.00% (Table S1; Figure 3b), respectively.

The mineral composition analysis of 14 samples was carried out to compare their relationship with the relative abundance of 17α(H)-diahopanes (Table S1). The results show that the studied core samples characterized by a very low content of carbonate mineral range from 0% to 11.4% with an average of 4.5%. Little pyrite was present in the all samples, indicating a weak bacteria sulfur reduction during those sediments deposition.

## 5 Discussion

### 5.1 Maturity on the Distribution of Diahopanes

The maximal sampled depth intervals of sediments from two wells are approximately 200 m, which do not make a difference to maturity. In addition, the *T*_max_ values and vitrinite reflectance (Table S1) indicate that these samples are mature.^42,43^ Therefore, the maturity appears to affect the relative abundance of 17α(H)-diahopane in this study area.

### 5.2 Organic Matter Input on the Distribution of Diahopanes

**Figure 4:**
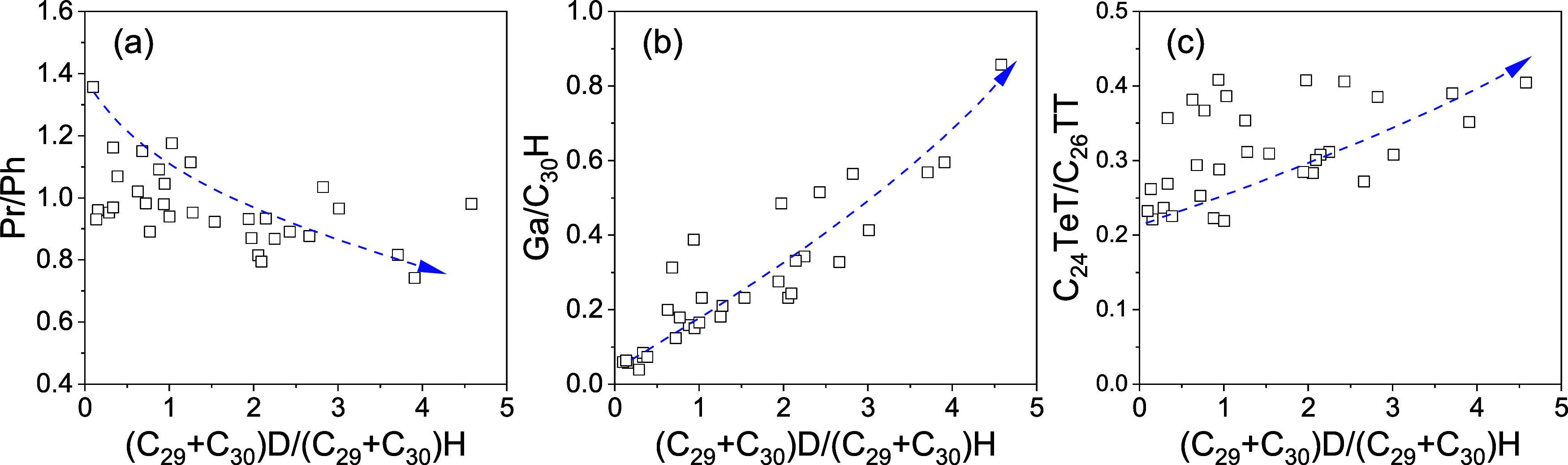
Cross plots of (C_29_ + C_30_)D/(C_29_ + C_30_)H vs (a) Pr/Ph, (b) Ga/C_30_H, and (c) C_24_TeT/C_2_TT showing the relative abundance of gammacerane and diahopanes. D: diahopanes; H: hopanes; Pr: pristane; Ph: phytane; Ga: gammacerance; TeT: tetracyclic terpene; and TT: tricyclic terpane.

The combination isoprenoids and *n*-alkanes are effective indicators for sedimentary environment and organic matter source determining, as well as the effects of maturation and biodegradation on those compounds.^44^ The distributions of *n*-alkanes and isoprenoids indicate (Figure S1; Table S1) that organic matter in those source rocks were attributed to the mixed input of aquatic organism and terrigenous higher plants (Figure 3a). Regular steranes are generally employed for the determination of organic matter input in sediments and crude oils.^45^ C_27_ sterane is dominant in source rock with major aquatic organism input while C_29_ sterane is predominant in that with terrigenous organic matter.^45,46^ Some specific biological species also can be contributed to C_29_ sterane.^5,46−50^ The C_27_–C_29_ regular steranes distributions of studied samples are similar and shaped a “V” pattern. The distributions of C_27_ and C_29_ regular steranes (Table S1; Figure 4b) also indicate a mixed input of aquatic organisms and terrigenous higher plants, which shows a small difference. This is consistent with the distribution of isoprenoids and *n*-alkanes (Figure 3a). Therefore, in this study, the organic matter source is similar in the selected samples and has a minor contribution to the different distributions of 17α(H)-diahopane.

### 5.3 Mineral Compositions on the Distribution of Diahopanes

It has been considered that clay minerals play a key role in the occurrence of arranged hopanes (e.g., Chen et al., 2015;^12^ Jiang et al., 2018^13^). Based on the mineral compositions in the cores from the study area (Table S1), clay minerals occurred in the cores with the highest abundance, followed by quartz and feldspar. Those data indicate a distinctly clastic sedimentation rather than carbonate, which consisted the depositional background and previous reports. Therefore, catalysis of clay likely is responsible for the formation of arranged hopanes in the sediments.

### 5.4 Sedimentary Environment on the Distribution of Diahopanes

Pristane (Pr) and phytane (Ph) are effective biomarkers for determining sedimentary environment of source rock and oil.^50,51^ The ratio of Pr/Ph < 1 in sediments is considered as a reducing water body, while those of Pr/Ph > 3 reply a distinctly oxidizing environment.^52^Figure 4a shows a negative correlation between the values of (C_29_ + C_30_ diahopanes)/(C_29_ + C_30_ hopanes) and Pr/Ph, indicating a control of sedimentary environment on the abundance of 17α(H)-diahopanes, whereby a reducing water body is helpful for the formation of those compounds. Abundant gammacerane occurring in the sediments implies a reducing and stratified water body. The relative abundances of 17α(H)-diahopanes show a good linear relationship with those of gammacerane (Figure 4b), which reflect that the sedimentary environment may be responsible for the differences of biomarker 17α(H)-diahopane distributions. In others words, a more saline environment may be in favor of the formation of 17α(H)-diahopane, which differs from the previous study.^28,30^

C_24_ tetracyclic terpane (C_24_Tet) has been identified in the sediments and crude oils and used for depositional environment definition and oil–oil as well as oil–source correlations. This compound has been considered to be a critical indicator for the reducing carbonate-evaporite water body.^53,54^Figure 4c illustrates the correlations between the relative abundance of diahopanes and that of C_24_Tet relative to C_26_TT. The result shows an increasing trend of (C_29_ + C_30_) dishopanes/(C_29_ + C_30_) hopanes values with the increasing of C_24_Tet/C_26_TT values, indicating the formation of diahopane compounds likely related to a reducing and evaporative environment.

Based on the distributions of 17-diahopanes and other biomarker distributions as well as mineral compositions in the selected cores, it indicates that high contents of clay and the sedimentary environment together control the abundance of 17-diahopanes. However, previous reports showed that an oxic or suboxic water body may favor for the formation of 17-diahopanes,^28−32^ while relatively high abundance of 17-diahopanes seems to be related to reducing water conditions in this study (Figure 4). This may be due to the different locations of the selected core samples deposited in the paleolake. The investigated core samples in this study likely deposited in the central area in the paleolake referring to a reducing water body, while the sediments shown by other authors were formed in the relatively edge zone of the paleolake corresponding to an oxidic environment. Therefore, the heterogeneity of sediments plays a role in the distributions of 17-diahopanes in this area.

## 6 Conclusions

In this work, we focus on the main controlling factors including maturity, organic matter source, and sediment environment for the distributions of 17α(H)-diahopane in the Ordos Basin. Maturity and organic matter source appear to have not been responsible for the relative content of 17-diahopanes. A similar maturity with a limited range in studied sediments suggests that the different abundances of 17α(H)-diahopane may not be attributed to the maturity. All the samples have like organic matter input with a mixture of aquatic organisms and terrigenous plants evidenced by the commensurate C_27_ and C_29_ regular steranes, which indicates that organic matter source have a minor contribute to the various 17α(H)-diahopane distributions. In summary, the mineral compositions and sedimentary environment have an effect on the distributions of 17-diahopanes in this area. A linearly positive relationship occurs between the relative abundance of 17α(H)-diahopane and gammacerane, indicating that a saline water condition appears to be helpful for the formation of 17α(H)-diahopane, but the internal mechanism remains unclear and should be further investigated. The heterogeneity of sediments plays a role in the distributions of 17-diahopanes in this area. Therefore, the biomarker 17α(H)-diahopane is an effective indicator related to the depositional environment and then for oil–oil/source correlation in this area.

## Supporting Information Available

Total ion currents showing the distributions of *n*-alkanes and isoprenoids and selected geochemical parameters of different samples (PDF)
