# Supplementary material for: Effect of Depositional Environment on the Occurrence of 17α(H)-Diahopanes in Triassic Lacustrine Sediments from the Ordos Basin, NW China
Source: ACS Omega. 2024 Nov 5;9(46):46057–63. doi: 10.1021/acsomega.4c06057 (PMC11579738; doi:10.1021/acsomega.4c06057)
Supplement: Supplementary file 1 — ao4c06057_si_001.pdf [file ao4c06057_si_001.pdf]

Supplementary material for

Effect of depositional environment on the occurrence of 17α(H)-diahopanes in Triassic lacustrine sediments from the Ordos Basin, NW China

Baohong Shi [a,b,*], Quansheng Liang [c], Erhu Liu [d], Xinyu Ai [e], Rong Wang [a,b]

[a] School of Earth Sciences and Engineering, Xi'an Shiyou University, Xi'an, Shaanxi, 710065, PR China

[b] Shanxi Key Laboratory of Petroleum Accumulation Geology, Xi'an Shiyou University, Shaanxi, 710065, China;

[c] Research Institute of Yanchang Petroleum (Group) Co. Ltd, Xi’an, Shaanxi, 710075, China

[d] Gas field Company, Shaanxi Yanchang (Group) Co. Ltd, Yan'an, Shaanxi, 716000, China

[e] Natural Gas Research Institute, Shaanxi Yanchang Petroleum (Group) Company Limited, Xi'an, Shaanxi 710075, China

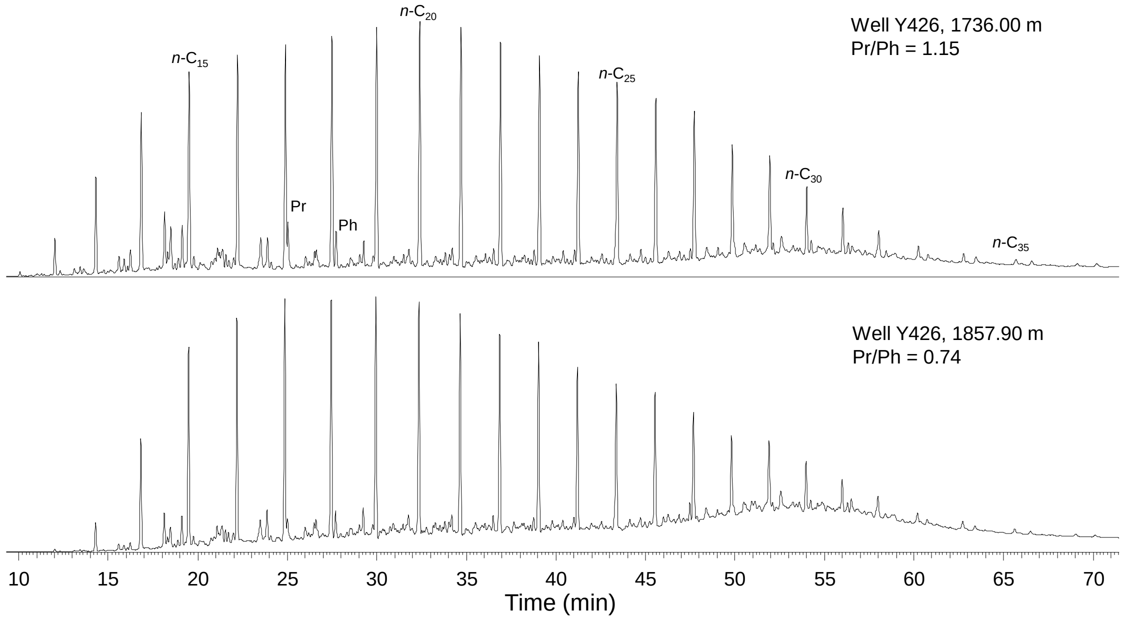

Fig. S1. Total ion currents (TIC) showing the distributions of n-alkanes and isoprenoids in the selected samples. Pr: pristane; Ph: phytane.

Table S1. Bulk geochemical and biomarker parameters of the core samples in this study.

Well	Depth	Lithology	TOC-pyrolysis data					Biomarkers				
			TOC	S1	S2	Tmax	Ro	C_{21-}/C_{22+}	CPI	OEP	Pr/Ph	C_{24}TeT/C_{26}TT
Y1011	1689.50	Black shale	5.75	3.32	18.13	453.00	0.79	2.34	1.10	1.04	0.88	0.27
Y1011	1702.15	Black shale	0.54	0.39	0.36	455.00	/	4.33	1.14	1.03	1.16	0.36
Y1011	1716.00	Black shale	4.93	8.87	26.13	450.00	0.81	2.77	1.29	1.06	0.82	0.22
Y1011	1732.18	Black shale	0.52	0.29	0.21	459.00	/	8.61	1.14	1.05	1.36	0.23
Y1011	1763.39	Black shale	8.80	3.86	24.51	454.00	0.82	2.51	1.12	1.04	0.76	0.22
Y1011	1772.33	Black shale	3.71	5.23	11.47	446.00	0.83	1.57	1.08	1.04	0.87	0.31
Y1011	1773.81	Black shale	5.53	6.19	16.05	445.00	0.85	1.68	1.09	1.04	0.93	0.31
Y1011	1774.31	Black shale	5.10	5.33	16.07	451.00	0.83	1.79	1.10	1.05	0.81	0.28
Y1011	1776.78	Black shale	4.11	4.20	11.80	449.00	0.81	1.64	1.09	1.04	0.79	0.30
Y1011	1778.00	Black shale	6.14	2.91	15.29	454.00	0.87	2.57	1.13	1.06	0.92	0.31
Y1011	1801.25	Dark grey mudstone	8.61	2.41	28.20	452.00	0.83	3.95	1.17	1.06	0.98	0.25
Y1011	1817.60	Dark grey mudstone	0.96	0.74	1.54	457.00	/	1.16	1.07	1.02	1.03	0.38
Y1011	1824.80	Black shale	1.95	0.83	4.37	456.00	/	2.99	1.15	1.05	1.04	0.29
Y1011	1831.10	Dark grey mudstone	0.81	1.16	1.59	448.00	/	1.67	1.10	1.04	0.98	0.41
Y1011	1840.60	Dark grey mudstone	1.40	1.08	2.32	454.00	/	1.64	1.10	1.05	1.11	0.35
Y1011	1850.90	Black shale	1.22	1.14	1.92	454.00	/	1.23	1.08	1.03	0.98	0.40
Y1011	1856.30	Black shale	1.03	1.94	2.08	450.00	/	1.20	1.08	1.03	0.82	0.39
Y1011	1860.40	Black shale	8.66	2.17	25.75	453.00	0.89	3.38	1.11	1.04	0.96	0.31
Y426	1718.20	Black shale	4.82	4.71	15.59	440.00	/	1.70	1.19	1.06	0.87	0.26
Y426	1736.00	Black shale	5.30	3.99	14.07	455.00	/	1.87	1.08	1.04	1.15	0.29
Y426	1743.90	Black shale	8.70	4.10	22.40	450.00	/	2.79	1.19	1.04	0.72	0.38
Y426	1743.95	Black shale	8.70	4.10	22.40	450.00	0.80	2.79	1.19	1.07	0.95	0.24
Y426	1746.50	Black shale	2.48	2.38	7.40	454.00	/	1.85	1.15	1.06	0.97	0.27
Y426	1757.35	Black shale	6.20	3.87	15.67	452.00	/	2.33	1.16	1.06	1.07	0.23
Y426	1763.79	Black shale	11.77	6.01	32.58	456.00	/	2.90	1.16	1.05	0.94	0.22
Y426	1775.12	Black shale	5.48	4.94	13.50	448.00	/	1.74	1.09	1.04	0.93	0.28
Y426	1793.97	Dark grey mudstone	2.78	1.04	7.08	454.00	/	2.44	1.12	1.05	0.95	0.31
Y426	1805.40	Black shale	1.02	2.87	2.11	447.00	/	1.19	1.11	1.05	0.89	0.37
Y426	1835.55	Dark grey mudstone	0.59	0.43	0.51	456.00	/	1.74	1.08	1.02	1.17	0.39
Y426	1844.10	Dark grey mudstone	0.87	0.50	1.21	456.00	0.91	1.23	1.06	1.01	0.89	0.41
Y426	1848.50	Dark grey mudstone	0.71	1.38	1.18	457.00	/	1.63	1.10	1.03	0.87	0.41
Y426	1857.90	Black shale	4.15	1.65	11.19	453.00	0.89	2.30	1.10	1.04	0.74	0.35

Note: TOC: Total organic carbon content, %; S1: volatile hydrocarbon (HC) content, mg/g TOC; S2: remaining (HC) generative potential, mg/g TOC; Tmax: temperature at maximum generation, °C; Ro: vitrinite reflectivity, %; C_{21-}/C_{22+}: C_{12}-C_{21}/C_{22}-C_{33} n-alkanes; Pr/Ph: Pristane/Phytane; C_{24}Tet/C_{26}TT: C_{24}Tetracyclic terpenes/C_{26} Tricyclic terpane

Well	Depth	Lithology	Biomarker					Mineral composition				
			D/H	G/H	C_{27}	C_{28}	C_{29}	Quartz	Feldspar	Carbonates	Pyrite	Clay minerals
Y1011	1689.50	Black shale	2.67	0.33	43.64	24.85	31.51	/	/	/	/	/
Y1011	1702.15	Black shale	0.34	0.07	29.24	23.77	47.00	/	/	/	/	/
Y1011	1716.00	Black shale	0.16	0.06	32.17	28.37	39.46	/	/	/	/	/
Y1011	1732.18	Black shale	0.10	0.06	31.36	23.56	45.09	16.8	7.5	6.8	0	68.9
Y1011	1763.39	Black shale	0.88	0.16	36.99	25.30	37.71	15.3	9.7	0	5	70
Y1011	1772.33	Black shale	2.25	0.34	42.03	25.73	32.24	17.8	11.3	0	0	70.9
Y1011	1773.81	Black shale	2.15	0.33	41.88	23.07	35.05	32.9	21.8	0	0	45.3
Y1011	1774.31	Black shale	2.06	0.23	42.73	26.00	31.26	28.8	21.4	8.1	0	41.7
Y1011	1776.78	Black shale	2.09	0.24	43.23	25.08	31.69	27.4	11.7	0	0	60.9
Y1011	1778.00	Black shale	1.54	0.23	35.84	26.25	37.91	25.2	12.1	0	0	62.7
Y1011	1801.25	Dark grey mudstone	0.73	0.12	37.38	29.75	32.87	/	/	/	/	/
Y1011	1817.60	Dark grey mudstone	2.83	0.56	41.11	12.12	46.77	/	/	/	/	/
Y1011	1824.80	Black shale	0.95	0.15	35.06	27.15	37.79	/	/	/	/	/
Y1011	1831.10	Dark grey mudstone	0.94	0.39	34.40	19.76	45.84	/	/	/	/	/
Y1011	1840.60	Dark grey mudstone	1.25	0.18	33.84	28.37	37.79	/	/	/	/	/
Y1011	1850.90	Black shale	4.58	0.86	40.14	22.00	37.87	/	/	/	/	/
Y1011	1856.30	Black shale	3.71	0.57	43.74	22.18	34.08	/	/	/	/	/
Y1011	1860.40	Black shale	3.02	0.41	36.89	28.50	34.61	/	/	/	/	/
Y426	1718.20	Black shale	0.14	0.06	31.13	27.93	40.94	/	/	/	/	/
Y426	1736.00	Black shale	0.68	0.31	47.77	21.24	31.00	/	/	/	/	/
Y426	1743.90	Black shale	0.63	0.20	37.20	23.86	38.95	/	/	/	/	/
Y426	1743.95	Black shale	0.29	0.04	34.13	29.40	36.47	/	/	/	/	/
Y426	1746.50	Black shale	0.34	0.08	34.96	27.99	37.06	/	/	/	/	/
Y426	1757.35	Black shale	0.39	0.07	34.00	28.68	37.32	/	/	/	/	/
Y426	1763.79	Black shale	1.01	0.16	36.12	25.92	37.96	/	/	/	/	/
Y426	1775.12	Black shale	1.94	0.27	40.65	24.36	34.99	32	11.7	0	0	56.3
Y426	1793.97	Dark grey mudstone	1.28	0.21	33.91	29.96	36.13	18.5	20.2	8.9	0	52.4
Y426	1805.40	Black shale	0.77	0.18	36.28	25.73	37.98	17.6	22	8.5	0	51.9
Y426	1835.55	Dark grey mudstone	1.03	0.23	37.06	22.62	40.32	27.6	19.7	8.9	0	43.8
Y426	1844.10	Dark grey mudstone	2.43	0.51	38.75	19.81	41.45	20.2	23.6	11.4	0	44.8
Y426	1848.50	Dark grey mudstone	1.98	0.48	38.02	23.91	38.07	28.8	24.8	10	0	36.4
Y426	1857.90	Black shale	3.91	0.59	35.85	28.56	35.59	31.7	20.2	0	0	48.1

Note: D/H: $(C_{29}+C_{30})$Diahopanes/$(C_{29}+C_{30})$Hopanes; G/H: Gammacerane/C_{30}Hopane; C_{27}: C_{27}/C_{27}-C_{29} regular steranes, %; C_{28}: C_{28}/C_{27}-C_{29} regular steranes, %; C_{29}: C_{27}/C_{27}-C_{29} regular steranes, %;
